# Clinical and genetic risk factors for Fulvestrant treatment in post-menopause ER-positive advanced breast cancer patients

**DOI:** 10.1186/s12967-018-1734-x

**Published:** 2019-01-15

**Authors:** Jingyu Liu, Jing Li, Hui Wang, Yikai Wang, Qiongzhi He, Xuefeng Xia, Zhe-Yu Hu, Quchang Ouyang

**Affiliations:** 1 The Affiliated Cancer Hospital of Xiangya School of Medicine, Central South University / Hunan Cancer Hospital, Changsha, 410013 China; 2 Department of Breast Cancer Medical Oncology, Hunan Cancer Hospital, No. 283, Tongzipo Road, Changsha, 410013 People’s Republic of China; 3 Key Laboratory of Translational Radiation Oncology of Hunan Province (2015TP1009), Changsha, 410013 China; 4 Department of Biostatistics and Bioinformatics, Emory University Rollins School of Public Health, Atlanta, 33022 USA; 5 Geneplus Beijing Institute, Beijing, 102206 China

**Keywords:** Post-menopause HR-positive advanced breast cancer, Fulvestrant, First-line, ctDNA *ERBB2* and *ESR1* mutation, Poor progress-free survival (PFS)

## Abstract

**Background:**

Among breast cancer (BC) patients, near 40% are post-menopause, and 70%–80% are hormone receptor (HR)-positive. About 30%–40% BC patients who are diagnosed as invasive carcinoma HR-positive BC would eventually develop metastatic breast cancers. In 2016, FALCON trial proves Fulvestrant as an effective first-line endocrine therapy for post-menopause HR-positive advanced BC (ABC) patients. But even after FALCON published, Fulvestrant is rarely used as first-line in real world ABC patients in China.

**Method:**

In this study, 136 Fulvestrant users were enrolled from 2015. To investigate the clinical and genetic risk factors for Fulvestrant treatment response in real world data, biostatistic and bioinformatic analysis tools were adopted.

**Result:**

KM curves showed that Fulvestrant first-line users had a median progression-free survival (mPFS) of 15.67 months, which was longer than the second-line users and third (or higher)-line users (mPFS = 7.47 and 5.43 months, respectively). 16 s (or higher)-line users were voluntarily received circulating tumor DNA (ctDNA) testing after progression. ctDNA testing results showed that compared to patients with PFS longer than 6 months, Fulvestrant users with PFS less than 6 months had a significantly higher mutation rate of *ESR1* or *ERBB2* gene (0/6 vs 6/10, Fisher’s Exact p-value = 0.03). Multivariate COX regression analysis showed that clinical features, including lymph node metastasis and HER-2 positive, were significant risk factors for poor PFS [hazard ratio (HR) = 2.396 and 2.863, respectively]; high portion of estrogen receptor-positive cells was significant protective factor (HR = 0.663). Propensity-score matching (PMS) analysis suggested that visceral metastasis, prior palliative chemotherapy, and old age at Fulvestrant usage were not significant influential factor for PFS.

**Conclusion:**

First-line Fulvestrant usage could guarantee a better prognosis than higher-line usage. *ESR1* or *ERBB2* mutation was found to be related to poor PFS in higher-line Fulvestrant users.

**Electronic supplementary material:**

The online version of this article (10.1186/s12967-018-1734-x) contains supplementary material, which is available to authorized users.

## Background

Breast cancer is the leading mortality cause for females worldwide [1]. The incidence rate of breast cancer increased significantly from 2000, and has become the most common malignant disease for females. Among breast cancer patients, near 40% are post-menopause, and 70%–80% are hormone receptor (HR)-positive. In women who present with early stage hormone receptor-positive breast cancer, metastatic disease eventually develops in up to 40%, although relapses may occur late, up to three decades after the initial diagnosis [2]. The goals of treatment for advanced breast cancer (ABC) patients include symptoms alleviation, quality of life (QOL) improvement, and survival extension [3]. To achieve this goal, endocrine therapy is an efficient treatment strategy to target estrogen receptor (ER), to block ER’s interaction with estrogen and to inhibit ER downstream pathways. Endocrine therapy is the first-line treatment regimen for ER-positive ABC patients [4], except for severe visceral conditions which need rapid control for the disease.

Fulvestrant, a selective ER degrader (SERD), is able to bind to ER, down-regulate ER protein and block ER function [5]. Based on an open-accessed, random and multi-center phase III clinical trial No. 0021, Fulvestrant has the similar efficiency with Anatrozole in post-menopause ER-positive ABC patients who fail in the first-line endocrine therapy; the median time-to-progression (TTP) is 5.4 months and 5.1 months in Fulvestrant group and Alatrozole group, respectively [hazard ratio (HR) = 0.98, 95% confidence interval (CI) = 0.8–1.21, p = 0.84] [5]. Both in vivo and in vitro studies prove that Fulvestrant inhibits ER transcription and promotes ER degradation in a dose-dependent manner [6–9]. Clinical research further confirms that Fulvestrant 500 mg is significantly superior to 250 mg [HR (95% CI) = 0.8 (0.68, 0.94), p = 0.006]; the median overall survival (OS) for Fulvestrant 500 mg group is 25.1 months, while the median OS for Fulvestrant 250 mg group is 22.8 months [10]. Thus, American Society of Clinical Oncology (ASCO) recommends Fulvestrant 500 mg to be the standard treatment for post-menopause ER-positive ABC patients who fails in the first-line endocrine therapy.

According to FALCON trial (a phase III clinical trial), Fulvestrant 500 mg is approved to be the first-line endocrine therapy for post-menopause ER-positive ABC patients. Fulvestrant 500 mg can significantly improve the PFS, compared with Anatrozole group [HR (95% CI) = 0.797 (0.637, 0.999), p = 0.0486]; the median PFS for Fulvestrant 500 mg group is 16.6 months, while the median PFS for Anatrozole group is 13.8 months [11]. But for visceral metastatic patients, the median PFS of Fulvestrant and Anatrozole users are 13.5 months and 15.9 months, respectively; in non-visceral metastatic patients, the median PFS for Fulvestrant users is 22.3 months (HR = 0.59, non-visceral metastatic subgroup vs visceral metastatic subgroup) [11]. This finding implies a poorer prognosis for visceral metastatic Fulvestrant users. Based on a multi-center retrospective analysis among ABC Fulvestrant users, patients with first-line usage, no prior palliative chemotherapy, and lower histology/nuclear grade have better prognosis; visceral metastasis have no significant effect on prognosis [12]. Some other opinions are obtained from Graham research findings; they support that patients with visceral metastasis benefit more from Fulvestrant treatment [13, 14].

In real world practice, except HR status and tumor burden, the drug sensitivity and anti-cancer efficiency are always the priority issues in clinical treatment decision [3]. Although Fulvestrant is a promising and well-tolerant anti-breast cancer drug, it still has drug-resistant problem like other endocrine drug [15]. Recent researches suggest that *PIK3CA* mutation, *ESR1* mutation, and ER/HER2 crosstalk are the underlying molecular mechanisms for Fulvestrant resistance [15–19]. Patients with these resistant-related events always have poor prognosis. Therefore, molecular evaluation before treatment would provide oncologists the clues for future drug sensitivity and prognosis. Circulating tumor DNA (ctDNA) testing is highly efficient in detecting *ESR1*, *PI3K* and *TP53* mutations in metastatic BC patients [20–22]. Due to the spatial heterogenicity of breast metastases, ctDNA testing is even more useful than tumor tissue genetic testing.

In this study, we aimed to collect real world data to retrospectively analyze the potential risk factors for Fulvestrant treatment where risk candidates included both clinical factors and genetic mutations.

## Materials and methods

### Patient cohort and clinical data collection

136 BC patients with relapse or metastases who received Fulvestrant treatment were enrolled in this study. These patients received treatment at the Department of Breast Cancer Medical Oncology in Hunan Cancer Hospital from June 2015 to August 2018. The study was approved by the Ethics Committee at the Hunan Cancer Hospital.

Informed consent was obtained from each patient prior to study onset. Specially, inclusion criteria includes: (1) cytologically or histologically confirmed AJCC stage IIIB–IV breast cancer patients, including locally unresectable ABC, relapsed and metastatic BC; (2) post-menopause patients; (3) based on ASCO definition, ER is positive and/or PR is positive; (4) according to RECIST 1.1 standard [23], patients had at least one detectable target lesion; (5) Performance score (PS) was 1–2 points. Excluding criterion were (1) multiple primary cancer patients; (2) premenopausal females; (3) Fulvestrant was administrated combined with other endocrine therapy, chemotherapy or targeting therapy.

Basic demographic and clinical information were collected, including age of primary BC diagnosis, age at Fulvestrant treatment, disease free survival (DFS), HR/human epidermal growth factor receptor 2 (HER2) status, TNM stage at primary BC diagnosis, treatment history at diagnosis (including primary BC surgery/radiation, chemotherapy, and endocrine therapy history), palliative treatment history after relapse or metastasis, and metastatic sites.

### Definition of progression and time-to-progression

The RECIST has established the guidelines for measurement of the tumoral targets and to assess the response to treatments. According to RECIST 1.1 standards, progression is defined as 20% increase in tumoral targets’ volume. In targeted therapy-based treatment trials of metastatic BC patients, PFS highly correlates with overall survival [24, 25]. Therefore, PFS was applied to evaluate the patient’s responses to Fulvestrant in this study.

Time to Treatment Failure (TTF) was the second measurement in this study. According to previous publication [12], TTF was defined as the period of time between the start and the termination of Fulvestrant treatment. If a patient was considered as disease progressed (PD), then the termination day was the day of progression; if a patient received palliative or replaced treatment, then the termination day was 28 days later to the last day of Fulvestrant treatment; if a patient requested to stop Fulvestrant and change to use other drugs, the termination day was 28 days later after last Fulvestrant treatment day or the start day of the next regimen, depending on which day was earlier; if a patient had PD, but the progression day could not be identified, then the termination day was the last Fulvestrant treatment day plus 28 days, or the start day of next regimen, depending on which day was earlier.

### Circulating tumor DNA testing

To investigate the genetic risk factors associated with Fulvestrant resistance and poor prognosis, ctDNA test was performed in 16 volunteers among enrolled 136 Fulvestrant users. The peripheral blood samples were collected and DNA extraction procedure followed the protocol as described previously [20]. Genomic DNA (gDNA) was sequenced as the normal control sample. Capture probes were designed to cover coding sequences and hot exons of 1021 genes that were frequently mutated in solid tumors. A detailed description of the capture experiments has been reported [20]. Single nucleotide variants (SNV) were called using MuTect (version 1.1.4) and NChot softwares [20]. Small insertions and deletions (Indels) were called using GATK. Somatic copy number alterations were identified with CONTRA (v2.0.8). Significant copy number variation was expressed as the ratio of adjusted depth between ctDNA and control gDNA. The final candidate variants were all manually verified in the Integrative Genomics Viewer.

### Propensity score match

A 1:1 propensity score matching (PSM) analysis was performed to reduce the potential bias between the two subgroups. To evaluate the effect of potential factors on Fulvestrant treatment without bias, propensity scores were calculated through logistic regression for each patient in compared subgroups. The covariates included in the logistic regression were all other clinical candidates except the PSM-evaluated one. Patients in each of the two groups were matched based on the propensity score. Covariate balance between each of the two groups was examined by Chi square test. Survival comparisons were then performed for the matched patients using the same methods as those in the unmatched patients.

### Statistical analyses

Numerical variables were summarized as the mean (standard deviation) and median (interquartile range). Categorical variables were reported as counts (percentage). An analysis of variance was used to compare continuous variables with symmetrical distributions across subgroups. Chi square tests and Fisher’s exact tests (n < 5) were used to compare categorical variables between subgroups. Mentel–Haenszel Chi square tests were used, when group number was more than two. Cox regression analysis was used to evaluate the univariate and multivariate risk of candidate risk factors for progression. Kaplan–Meier (KM) curves were used to plot survival distributions against progression, and the log-rank test was used to assess differences in PFS among subgroups. A receiver operating characteristic (ROC) curve was then calculated to determine the optimal cutoff of the age at Fulvestrant usage that maximized sensitivity and specificity in predicting a better PFS. All tests of hypotheses were two-tailed and conducted at a significance level of 0.05, and at a marginal significance level of 0.15. Statistical analyses were conducted using SAS 9.4.

## Results

### Demographic and clinical features

136 Fulvestrant users were enrolled in this study. 17 (12.5%) patients received Fulvestrant as the first-line endocrine treatment. 61 (44.85%) patients received Fulvestrant as the second-line endocrine treatment. 58 (42.65%) patients received Fulvestrant as the third or even later lines of endocrine treatment. Patients with Fulvestrant at later (≥ third) line were younger at diagnosis and Fulvestrant usage, and had shorter DFS (Table 1). Moreover, more late users had surgery menopause, visceral metastasis, and palliative chemotherapy (Table 1). Other clinical features, such as HR/HER2 status, nuclear or histological grade, stage, menupause method, primary treatment history, bone and lymph node metastasis were not significantly distinct among Fulvestrant early users and late users.

**Table 1:** Clinical characteristics of ER-positive patients with Fulvestrant usage (n = 136)

Covariates	Level	All patients (n = 136)	Fulvestrant usage			p-value^†^
			First line (n = 17)	Second line (n = 61)	≥ Third line (n = 58)	
Age at diagnosis (years)		46.67 ± 9.66, 46 (39, 55)	48.26 ± 8.62, 48.5 (39.5, 56)	48.19 ± 10.04, 47 (39, 58)	44.69 ± 9.07, 44 (39, 50)	0.07
Age at FX usage (years)		53.37 ± 9.52, 53 (47, 62)	56.63 ± 7.16, 55.5 (52.5, 63)	54.63 ± 9.74, 55.5 (47, 62)	51.19 ± 9.27, 51 (45, 64)	0.01
Weight		58.67 ± 12.37, 58 (54, 62)	56.33 ± 2.88, 56.5 (55, 58)	57.21 ± 7.73, 57 (53, 61)	60.10 ± 15.94, 58 (54, 62)	0.27
ER	Negative	2 (1.47%)	0 (0%)	2 (3.28%)	0 (0%)	0.90
	1%–10%	3 (2.21%)	0 (0%)	1 (1.64%)	2 (3.45%)	
	10%–50%	11 (8.09%)	0 (0%)	6 (9.84%)	5 (8.62%)	
	50%–80%	37 (27.21%)	5 (29.41%)	16 (26.23%)	16 (27.59%)	
	80%–100%	25 (18.38%)	5 (29.41%)	10 (16.39%)	10 (17.24%)	
	Positive unknown %	58 (42.65%)	7 (41.18%)	26 (42.62%)	25 (43.10%)	
PR	Negative	18 (13.24%)	3 (17.65%)	6 (9.84%)	9 (15.52%)	0.52
	Positive	118 (86.76%)	14 (82.35%)	55 (90.16%)	49 (84.48%)	
HER2	Positive	13 (9.56%)	3 (17.65%)	3 (4.92%)	7 (12.07%)	0.17
	Negative	123 (90.44%)	14 (82.35%)	58 (95.08%)	51 (87.93%)	
Nuclear or histological grade	2	35 (25.74%)	3 (17.65%)	18 (29.51%)	14 (24.14%)	0.84
	3	51 (37.50%)	6 (35.29%)	22 (36.07%)	23 (39.66%)	
	Unknown	50 (36.76%)	8 (47.06%)	21 (34.43%)	21 (36.21%)	
Stage	I	15 (11.03%)	1 (5.88%)	5 (8.20%)	9 (15.52%)	0.35
	II	35 (25.74%)	5 (29.41%)	17 (27.87%)	13 (22.41%)	
	III	53 (38.97%)	4 (7.55%)	26 (42.62%)	23 (39.66%)	
	IV	2 (1.47%)	1 (5.88%)	1 (1.64%)	0 (0%)	
	Unknown	31 (22.79%)	6 (35.29%)	12 (19.67%)	13 (22.41%)	
Menopause	Natural menupause	83 (61.03%)	13 (76.47%)	41 (67.12%)	29 (50.00%)	0.02
	OFS (OFS + surgery)	24 (17.65%)	4 (23.53%)	10 (16.39%)	10 (17.24%)	
	Surgery	29 (21.32%)	0 (0%)	10 (26.39%)	19 (32.76%)	
Treatment of primary diagnosis	Primary site surgery	134 (98.53%)	17 (100%)	60 (98.36%)	57 (98.28%)	0.49
	Primary site radiation	52 (38.24%)	5 (29.41%)	23 (37.70%)	24 (41.38%)	0.67
	Chemotherapy	117 (86.03%)	14 (82.35%)	52 (85.25%)	51 (87.93%)	0.78
	Endocrine therapy	100 (73.53%)	13 (76.47%)	40 (65.57%)	47 (81.03%)	0.15
Treatment after relapse or metastasis	Radiation	21 (15.44%)	0 (0%)	10 (16.39%)	11 (18.97%)	0.15
	Chemotherapy	80 (58.82%)	6 (35.29%)	28 (45.90%)	46 (79.31%)	< 0.0001
DFS (years)*		4.86 ± 3.38, 4.24 (2.48, 6.96)	8.28 ± 3.94, 7.93 (5.89, 10.64)	5.01 ± 3.21, 4.69 (2.84, 7.36)	3.61 ± 2.57, 3.07 (2.00, 5.10)	< 0.0001
Metastatic sites	Lymph nodes	53 (38.97%)	10 (58.82%)	24 (39.34%)	19 (32.76%)	0.17
	Bone	93 (68.38%)	8 (47.06%)	45 (73.77%)	40 (68.97%)	0.12
	Visceral	68 (50.00%)	6 (35.29%)	30 (49.18%)	32 (55.17%)	0.01

* DFS indicated the time from diagnosis of BC to the diagnosis time of relapse or metastasis. For ^†^ p-value calculation, ANOVO analysis was used to compare continuous variables with symmetrical distributions across subgroups, Mentel–Haenszel Chi square tests and Fisher’s exact tests (n < 5) were used to compare categorical variables between subgroups

### Clinical risk factors for poor treatment response

To evaluate the risk factors for poor response in Fulvestrant users, univariate COX regression analyses were performed. As shown in Table 2, compared to the first-line usage, the second-line treatment was a marginal risk factor for disease progression (HR [95% CI] = 1.911 [0.803, 4.547], p = 0.14) and treatment failure (TTF) (HR [95% CI] = 2.008 [0.849, 4.745], p = 0.11); compared to the first-line usage, the late Fulvestrant usage (≥ third line) was a significant risk factor for both disease progression (HR [95% CI] = 2.420 [1.026, 5.711], p = 0.04) and treatment failure (HR [95% CI] = 2.668 [1.135, 6.272], p = 0.03). Other significant risk factors included HER2-positive status, higher nuclear/histological grade, advanced BC stage at diagnosis, and lymph node metastasis at cancer relapse or metastasis.

**Table 2:** Univariate COX regression analysis for the risk factors for progression (PFS) and time-to-failure (TTF) in Fulvestrant users

Covariates	Level	PFS		TTF	
		Hazard ratio (95% CI)	p-value	Hazard ratio (95% CI)	p-value
Fulvestrant usage	First line	Ref		Ref	
	Second line	1.911 (0.803, 4.547	0.14	2.008 (0.849, 4.745)	0.11
	≥ Third line	2.420 (1.026, 5.711)	0.04	2.668 (1.135, 6.272)	0.03
Age at diagnosis (years)		1.001 (0.977, 1.026)	0.94	1.004 (0.981, 1.028)	0.72
Age at FX usage (years)		0.990 (0.966, 1.015)	0.44	0.994 (0.971, 1.017)	0.60
	< 62	Ref		Ref	
	≥ 62	0.753 (0.441, 1.288)	0.30	0.714 (0.425, 1.199)	0.20
Weight		0.999 (0.982, 1.016)	0.37	0.997 (0.979, 1.015)	0.75
DFS (years)		0.950 (0.886, 1.019)	0.15	0.948 (0.887, 1.013)	0.11
ER	Negative	0.610 (0.128, 2.903)	0.53	0.795 (0.168, 3.763)	0.77
	1%–50%	Ref		Ref	
	50%–100%	0.693 (0.318, 1.510)	0.36	0.869 (0.404, 1.869)	0.72
	Unknown	0.706 (0.326, 1.528)	0.71	0.894 (0.417, 1.916)	0.77
PR	Negative	Ref		Ref	
	Positive	1.171 (0.602, 2.277)	0.64	1.073 (0.582, 1.981)	0.82
HER2	Negative	Ref		Ref	
	Positive	2.024 (0.997, 4.109)	0.05	1.833 (0.936, 3.587)	0.08
Nuclear or histological grade	2	Ref		Ref	
	3	1.683 (0.942, 3.004)	0.08	1.476 (0.861, 2.530)	0.15
	Unknown	1.410 (0.782, 2.541)	0.25	1.219 (0.694, 2.139)	0.49
Stage at BC diagnosis	0/I	Ref		Ref	
	II	1.476 (0.791, 2.755)	0.22	1.378 (0.759, 2.503)	0.29
	III/IV	1.709 (1.001, 2.919)	0.05	1.607 (0.969, 2.666)	0.07
Menopause	Natural menupause	Ref		Ref	
	OFS (OFS + surgery)	0.850 (0.463, 1.560)	0.60	0.697 (0.384, 1.265)	0.24
	Surgery	1.145 (0.673, 1.948)	0.62	1.049 (0.624, 1.764)	0.86
Treatment of primary diagnosis	Primary site surgery	0.807 (0.112, 5.837)	0.83	0.515 (0.126, 2.107)	0.36
	Primary site Radiation	1.075 (0.688, 1.682)	0.75	1.133 (0.738, 1.740)	0.57
	Chemotherapy	0.995 (0.495, 2.003)	1.00	0.968 (0.499, 1.881)	0.92
	Endocrine therapy	1.223 (0.729, 2.049)	0.45	1.263 (0.771, 2.068)	0.35
Treatment after relapse or metastasis	Radiation (Yes vs No)	1.091 (0.589, 2.022)	0.78	0.879 (0.476, 1.621)	0.68
	Chemotherapy (Yes vs No)	1.394 (0.873, 2.226)	0.16	1.413 (0.905, 2.206)	0.13
Metastatic sites	Lymph nodes	1.623 (1.041, 2.531)	0.03	1.509 (0.986, 2.311)	0.06
	Bone	1.096 (0.682, 1.762)	0.83	1.180 (0.745, 1.869)	0.57
	Visceral	1.036 (0.671, 1.601)	0.87	0.967 (0.637, 1.468)	0.87

To avoid confounding effects, multivariate COX regression analysis was performed. As shown in Additional file 1: Table S1, compared to the first-line usage, both the second-line and ≥ third-line usages were risky for progression and treatment failure in multivariate COX model. HER2-positive was risk for progression (HR = 2.396, p = 0.04), while 50%–100% ER-positive was protective for progression (HR = 0.663, p = 0.02). In multivariate COX model, advanced stage at diagnosis and lymph node metastases were also significant risk factors for progression and treatment failure. Interestingly, older age at Fulvestrant usage was a significant protective factor in multivariate COX model (HR = 0.88, p = 0.03, Additional file 1: Table S1).

### Survival analysis for patients stratified by significant candidates

**Fig. 1:**
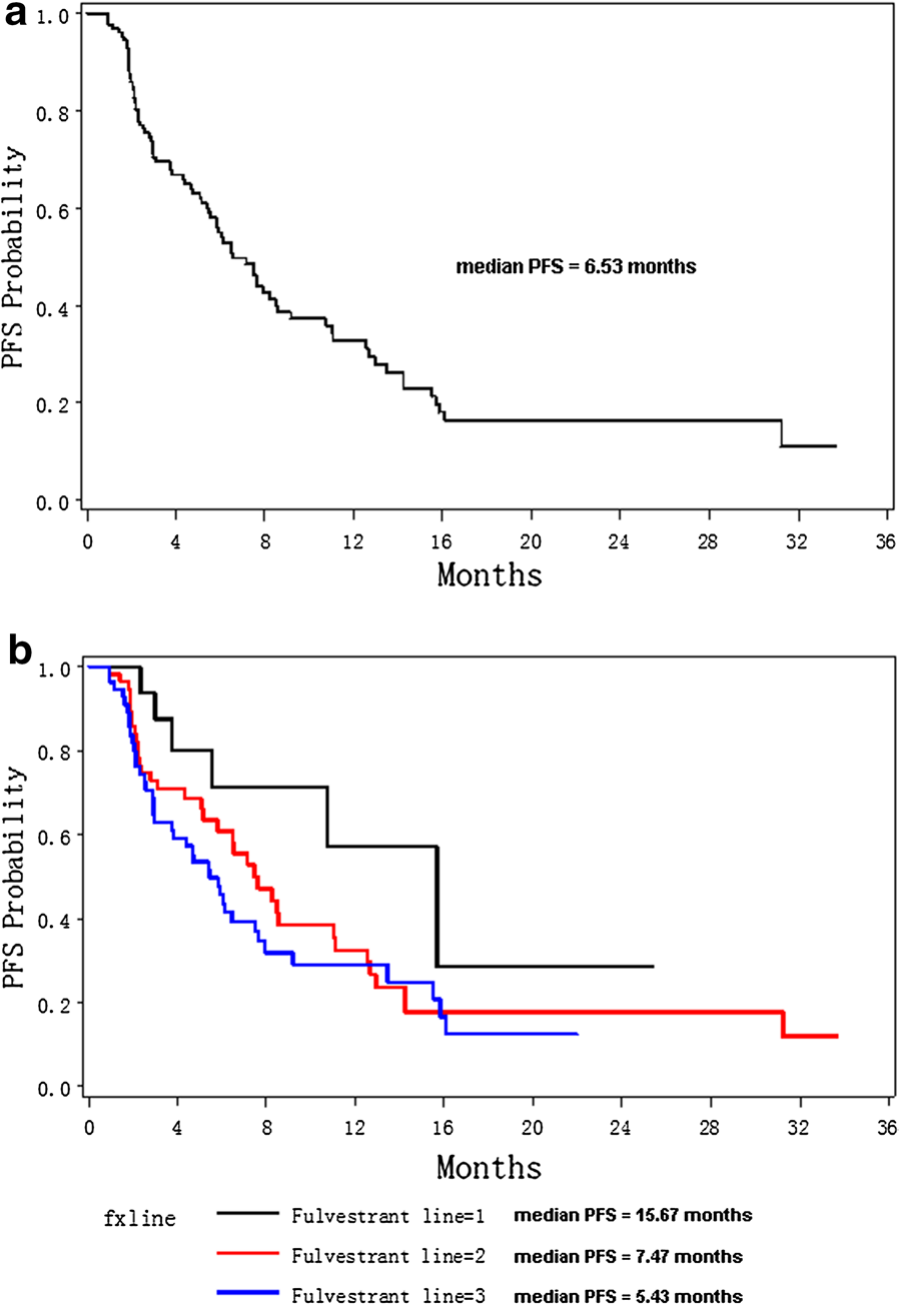
Kaplan–Meier curves for progression-free survival probabilities for all enrolled 136 patients (**a**) and stratified by Fulvestrant lines (**b**)

**Fig. 2:**
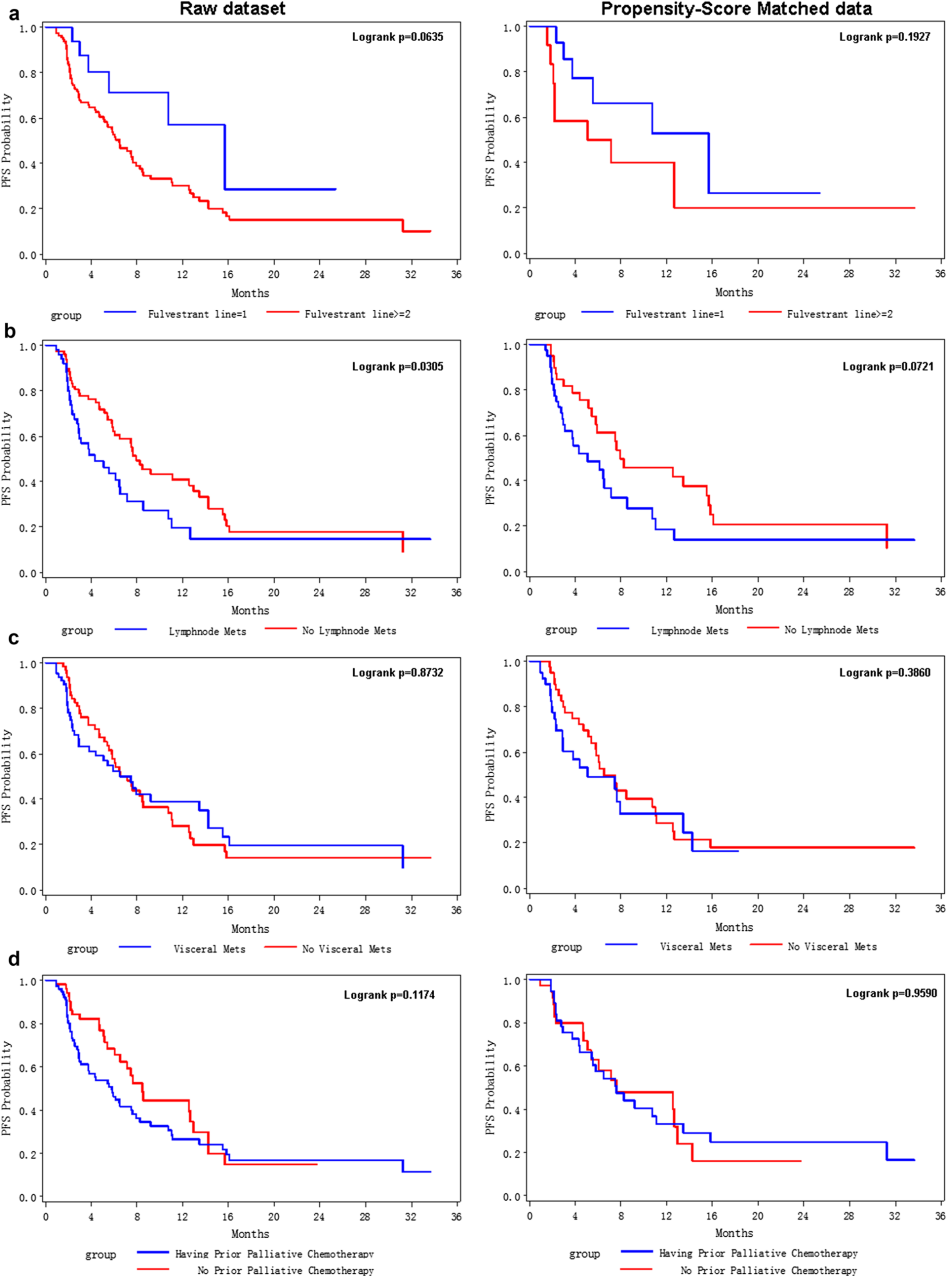
Kaplan–Meier curves for progression-free survival probabilities stratified by Fulvestrant lines (**a**), lymph node metastasis (**b**), visceral metastasis (**c**), and prior palliative chemotherapy (**d**). The left panel was derived from enrolled raw dataset; the right panel showed KM curves for propensity score-matched samples

To further evaluate the effect of above significant factors on Fulvestrant users, lifetest and PSM analyses were conducted. As shown in Fig. 1, the median PFS for all enrolled patients was only 6.53 months. But, patients with Fulvestrant first-line treatment had longer mPFS (15.67 months), which was much longer than Fulvestrant second-line users (mPFS = 7.47 months) and patients with Fulvestrant line ≥ 3 treatment (mPFS = 5.43 months). Lifetest showed a significant better PFS for first-line users than second (or above)-line users (p-value = 0.0635, Fig. 2a left). In PSM data (right panel of Fig. 2a), p-value was not significant due to small sample size (a total of 30 samples after PS matched), but the Kaplan-Merier (KM) curve of first-line Fulvestrant users (blue line) was distinctively higher than the KM curve of higher line users (red line).

Lymph node metastasis was a significant risk factor for poor PFS in both univariate and multivariate COX model; PSM analysis further confirmed this effect (Fig. 2b, p = 0.07 in PSM data). However, visceral metastasis did not show a significant risk effect on PFS for Fulvestrant users in both raw data and PSM data (Fig. 2c). As for prior palliative chemotherapy before Fulvestrant treatment, it showed a marginal risk effect for PFS in KM curve analysis (Fig. 2d left panel, logrank p = 0.11) and univariate COX analysis (p = 0.16, Table 2); but in PSM data, the stratified KM curves were most the same (Fig. 2d right panel, logrank p = 0.96). Also, because patients with prior palliative chemotherapy were all second-line or higher-line Fulvestrant users (Table 1), prior palliative chemotherapy showed a marginal risk effect on PFS (Table 2); when PSM removed the unbalanced distribution of patients with prior palliative chemotherapy among distinct lines of Fulvestrant users, the risk effect of prior palliative chemotherapy disappeared (Fig. 2e).

### ctDNA mutations related to Fulvestrant resistance and poor prognosis

**Fig. 3:**
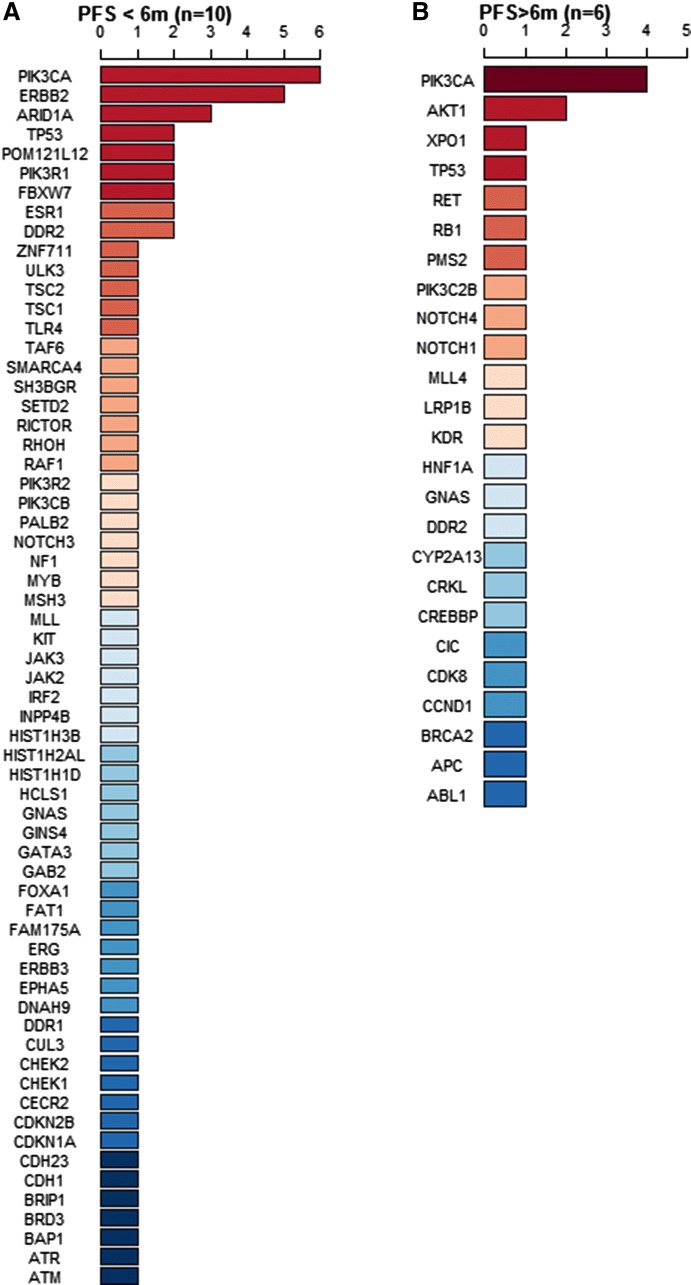
Circulating tumor DNA (ctDNA) gene mutation profiles in 16 volunteer Fulvestrant users, stratified by PFS lengths, PFS < 6 months (**a**) and PFS > 6 months (**b**). Dark red represents the most common mutated genes and dark blue represents the rarest mutations. If the mutated genes appeared at the same frequency, they are ranked in alphabetic order

**Fig. 4:**
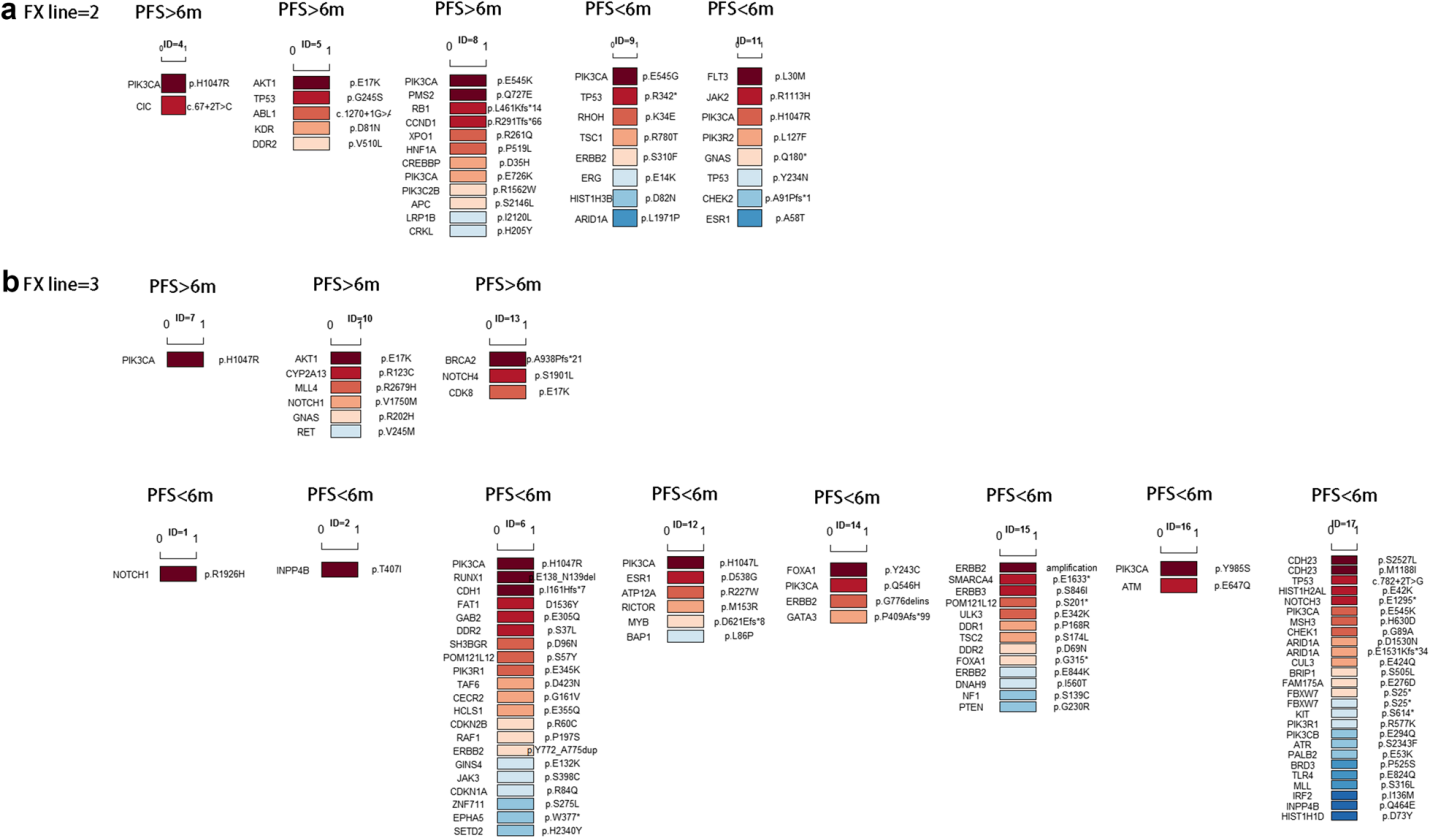
ctDNA gene mutations in 16 individual Fulvestrant users. 5 patients received second-line Fulvestrant treatment (**a**), and 11 patients received third-line or higher line Fulvestrant treatment (**b**). Dark red represents the most frequent mutated genes and dark blue represents the rarest mutations. If the mutated genes appeared at the same frequency, they are ranked in alphabetic order

To explore the molecular basis of Fulvestrant resistance, 16 Fulvestrant second- or higher-line users participated ctDNA testing. According to their PFS lengths, these patients were divided into two subgroups: PFS < 6 months and PFS > 6 months. Figure 3 ranked the mutated genes. If 1 patient had two mutations within in one gene (e.g., patient ID = 8 had two PIK3CA mutations, p.E545K and p.E726K, Fig. 4a), this gene would be counted twice in Fig. 3. As shown in Fig. 3, in patients with poor prognosis (PFS < 6 months), *TP53*, *ERBB2* and *ESR1* gene mutations were the top frequent mutations; other genes, including *ARD1A*, *FBXW7*, *DDR2*, ect, were also frequent mutations. For patients with PFS longer than 6 months, no *ERBB2* and *ESR1* gene mutations were detected.

To explore the correlation of *TP53*, *PIK3CA*, *ERBB2* and *ESR1* mutations with poor PFS, fisher’s exact test was used to compare the patients’ gene mutation frequencies between two PFS subgroups. Here, if 1 patient had mutations in one gene, no matter how many mutations were there in 1 patient, only 1 patient is counted for frequency comparison. As shown in Table 3, when compared to PFS > 6 months subgroup, PFS < 6 months subgroup had significantly higher mutation frequency of *ESR1* or *ERBB2* mutation (p = 0.03). In addition, marginally more patients with PFS < 6 months had *TP53*, *ESR1* or *ERBB2* mutations (p = 0.011).

**Table 3:** ctDNA gene mutations of 16 Fulvestrant second- or higher-line users

Covariate	Overall (N = 16)	PFS subgroups		
		PFS < 6 months (n = 10)	PFS > 6 months (n = 6)	p-value*
PIK3CA	10 (62.50%)	7 (70.00%)	3 (50.00%)	0.61
TP53	4 (25.00%)	3 (30.00%)	1 (16.67%)	1.00
ESR1	2 (12.50%)	2 (20.00%)	0 (0%)	0.50
ERBB2	4 (25.00%)	4 (40.00%)	0 (0%)	0.23
ESR1/ERBB2	6 (37.50%)	6 (60.00%)	0 (0%)	0.03
TP53/ESR1/ERBB2	8 (50.00%)	7 (70.00%)	1 (16.67%)	0.11

According to their PFS lengths, these patients were divided into two subgroups: PFS < 6 months and PFS > 6 months

* p-values were calculated by using Fisher’s exact tests (n < 5) for categorical variables comparison between PFS < 6 months group and PFS > 6 months group

### Fulvestrant resistance were related to *ESR1* mutation or *ERBB2* mutation

Among 16 Fulvestrant users with ctDNA testing, five received the second-line Fulvestrant treatment, and 11 patients received ≥ third-line Fulvestrant treatment (Additional file 2: Figure S1). In second-line Fulvestrant users, PFS of 2/5 (40%) patients were less than 6 months (ID = 9 and ID = 11), while PFS of the rest 3/5 (60%) patients were longer than 6 months (ID = 4, 5, 8, Fig. 4a). In ≥ third-line Fulvestrant users, PFS of 8/11 (73%) patients were less than 6 months, and PFS of 3/11 (27%) patients were longer than 6 months (ID = 7, 10, 13, Fig. 4b). In all 10 PFS < 6 months patients, two had *ESR1* missense mutation (ID = 11 p.A58T and ID = 12 p.D538G), 4 patients had *ERBB2* mutation (ID = 6 p.Y772_A775dup; ID = 9 p.S310F; ID = 14 p.G776delins; ID = 15 had both *ERBB2* amplification and *ERBB2* p.E844K missense mutation Fig. 4b), and 3 patients had *TP53* mutation (ID = 9, 11 and 17). In all 6 PFS > 6 months patients, none of them had *ESR1* or *ERBB2* mutations.

## Discussion

In this study, we retrospectively analyzed the clinical factors that might influence the treatment response of advanced ER-positive breast cancer patients to Fulvestrant. We also utilized ctDNA testing to investigate Fulvestrant-related drug resistant mutations. By using COX regression analysis, we found that Fulvestrant first-line treatment, older age at Fulvestrant usage, and high ER-positive percentage were protective factors for PFS in Fulvestrant users. But, ≥ second-line Fulvestrant usage, HER2-positive, higher nuclear or histological grade, late stage (stage III/IV) at BC diagnosis, and lymph node metastases were important risk factors for poor PFS prognosis for Fulvestrant users (Table 2 and Additional file 1: Table S1). PSM analysis further confirmed the risk effect of late-lines treatment and lymph node metastasis (Fig. 2).

Currently, Fulvestrant is recommended as the standard first-line endocrine therapy for post-menopause ER-positive advanced BC patients. But, based on our study, only 17/136 (12.5%) post-menopause ER-positive ABC patients received first-line Fulvestrant treatment, 61/136 (44.85%) patients received second-line Fulvestrant treatment, and 58/136 (42.65%) patients received third-line (and higher lines) Fulvestrant treatment. The median PFS for first-line, second-line and third-line (including higher lines) users were estimated to be 15.67 months, 7.47 months and 5.43 months, respectively (Fig. 1). Therefore, the earlier Fulvestrant is used, the better prognosis patients would have. In addition, compared to the ER status at diagnosis or surgery biopsy, ER status would change as disease progression in later stage. Drug resistance would also appear after multi-line endocrine therapies. Therefore, based on the findings of this study, we recommended ER-positive ABC patients to receive Fulvestrant treatment as early as possible. Multivariate COX regression analysis suggested high ER-positive percentage (50–100%) to be a beneficial factor for prognosis (Additional file 1: Table S1). This was consistent with clinical observations that patients with high ER level were more sensitive to endocrine therapy and thus the prognosis would be better.

Univariate COX analysis showed prior palliative chemotherapy as a marginal risk factor for poor prognosis. The median PFS for Fulvestrant users who had no prior palliative chemotherapy was 8.47 months, compared to patients with prior palliative chemotherapy with median PFS of 5.8 months (logrank p-value = 0.1174, Fig. 2d). Usually, patients with rapid tumor growth and symptomatic visceral metastases were firstly treated with palliative chemotherapy, because these patients needed rapid control for the disease. Thus, Fulvestrant was used as second-line or even higher-line regimen for these patients. Undoubtedly, these patients had poor response for late-line Fulvestrant treatment. When PSM was used to evaluate prior palliative chemotherapy, none first-line users were included; all PSM patients for prior palliative chemotherapy were second-line or higher-line patients; in this case, patients with prior palliative chemotherapy had no significant different PFS from patients with other prior palliative drug treatment.

According to current clinical researches, no report about the relationship between lymph node metastases and PFS prognosis has been demonstrated in Fulvestrant users. In our study, both COX regression analysis and PSM analysis showed a significant risk effect of lymph node metastases against PFS in Fulvestrant users (Fig. 2b). But, these results need to be carefully interpreted. More well-designed perspective studies are required to confirm our results.

As for visceral metastasis, it is another important clinical event for evaluation. FALCON trial has suggested that non-visceral metastasis patients benefit more from Fulvestrant [11]. However, Kawaguchi H reports that visceral metastasis is irrelevant to PFS in Fulvestrant users [12], which is similar to our findings. We suppose that distinct enrollment criterion lead to such a discrepancy. The enrolled patients in FALCON trial are ER-positive/HER2-negative patients without any prior endocrine therapy. But in our retrospective study, most (73.53%) patients had received prior adjuvant endocrine therapy after surgery (Table 1). After long-term treatment, our patients more or less had intrinsic or required drug-resistance, so their treatment response to Fulvestrant would be inferior to the patients in FALCON trial. In addition, invisible visceral micro-metastasis might be another reason for such discrepancy. As we known, micro-metastasis sometimes exists even after mastectomy surgery and adjuvant chemotherapy [26, 27]; but current clinical tools cannot detect these micro-metastases, especially for visceral micro-metastases. Therefore, inadequate evaluation for disease status in ABC patients would lead to insufficient treatment and rapid tumor progression.

Till now, no research about ctDNA gene mutations has been reported in Fulvestrant users. Therefore, in this study, we recruited 16 volunteers to screen their ctDNA profile when disease progressed after Fulvestrant treatment. Although the sample size was relatively small, but we still obtain some important clues for the molecular basis about Fulvestrant resistance. All recruited 16 patients received second- or higher-line Fulvestrant treatment. By using ctDNA testing, *PIK3CA* was found to be the most common (62.5%) mutated gene in these 16 patients. *PIK3CA* mutated patients with *ESR1* or *ERBB2* mutation had the shortest PFS. As shown in Table 3, 60% patients in PFS < 6 months group had *ESR1* or *ERBB2* mutation. Two *ESR1* mutated patients (ID = 11 and ID = 12) were both ABC patients with prior aromatase inhibitors (AI) treatment. In ER-positive patients, *ESR1* mutation is not only associated to AI treatment failure [28], but also induces drug resistance to Fulvestrant [19]. The crosstalk between PI3K and ER pathway and between PI3K/ERBB2 pathway also cause patients’ resistance to endocrine therapy [17]. In COX regression analysis, HER2-positive status was also confirmed as a risk factor for poor prognosis (Table 2 and Additional file 1: Table S1).

There were some limitations in this study. First, in this single-center retrospective study, the sample size was relatively small, and we lacked drug efficiency comparison of Fulvestrant with other endocrine drugs. Second, in this retrospective observational study, none first-line Fulvestrant user received ctDNA testing. So, we could not get information about the mutation situation in first-line users. In fact, only 16 Fulvestrant second- or higher-line users received ctDNA testing. It was good for us to detect the ctDNA profile in treatment failure patients, but no dynamic ctDNA surveillance during Fulvestrant treatment was obtained for us to find other potential resistance-related mutations. In further investigation, we will continue to focus on Fulvestrant first line users and conduct ctDNA testing.

In recent years, clinical researches have made great effort to realize individualized treatment by optimizing clinical settings and targeting specific biological features [29]. Currently, anti-breast cancer therapy has entered an era of precision medication [30]. A double-blinded phase III clinical trial PALOMA-3 investigate the efficiency of Palbociclib plus Fulvestrant vs Fulvestrant alone in HR+/HER2− post-menopause patients with prior endocrine therapy failure; compared to Fulvestrant alone group, Palbociclib plus Fulvestrant shows a better prognosis [31]. Another random double-blinded placebo-controlled phase II clinical trial (PrE0102) shows that mTOR inhibitor Everolimus could improve Fulvetrant treatment outcome in AI-resistant ER-positive metastatic breast cancer (MBC) patients [32]; compared to Everolimus alone, Fulvestrant plus Everolimus could significantly prolong median PFS from 5.1 to 10.3 months [HR (95% CI) = 0.61 (0.40, 0.92), p = 0.02]. In addition, MONALEESA-3 trial proves that Fulvestrant has good treatment efficiency in both single drug treatment and combined regimen [33–35]. In future, more large-scale perspective research plus ctDNA surveillance would provide more useful clinical and genetic information about Fulvestrant treatment. Breast cancer patients would have more survival opportunity by using Fulvestrant-based combined targeting therapy.

## Conclusion

First-line Fulvestrant usage could guarantee a better prognosis than higher-line usage. Fulvestrant first-line users had a median PFS of 15.67 months, which was longer than the second-line users and third (or higher)-line users (mPFS = 7.47 and 5.43 months, respectively). COX regression analysis showed that lymph node metastasis and HER-2 positive were significant risk factors for poor PFS; high ER-positive was a significant protective factor. In addition, *ESR1* or *ERBB2* mutation was found to be related to poor PFS in higher-line Fulvestrant users.

## Additional files

**Additional file 1: Table S1.** Multivariate COX regression analysis for the risk factors for progression (PFS) and time-to-failure (TTF) in Fulvestrant users.

**Additional file 2: Figure S1.** ctDNA gene mutation profiles in Fulvestrant users, stratified by Fulvestrant lines, second-line users (**A**), and third or higher-line users (**B**). Dark red represents the most common mutated genes and dark blue represents the rarest mutations. If the mutated genes appeared at the same frequency, they are ranked in alphabetic order.

## Abbreviations

BC: breast cancer
HR: hormone receptor
ABC: advanced breast cancer
mPFS: median progression-free survival
PFS: progression-free survival
ctDNA: circulating tumor DNA
HR: hazard ratio
ER: estrogen receptor
QOL: quality of life
SERD: selective ER degrader
TTP: time-to-progression
CI: confidence interval
OS: overall survival
ASCO: American Society of Clinical Oncology
DFS: disease free survival
HER2: human epidermal growth factor receptor 2
gDNA: genomic DNA
SNV: single nucleotide variants
Indels: insertions and deletions
PSM: propensity score matching
KM: Kaplan–Meier
ROC: receiver operating characteristic
TTF: time to treatment failure
MBC: metastatic breast cancer
