# Supplementary material for: Clinical and genetic risk factors for Fulvestrant treatment in post-menopause ER-positive advanced breast cancer patients
Source: J Transl Med. 2019 Jan 15;17:27. doi: 10.1186/s12967-018-1734-x (PMC6334389; doi:10.1186/s12967-018-1734-x)
Supplement: Supplementary file 1 — Additional file 1: Table S1. Multivariate COX regression analysis for the risk factors for progression (PFS) and time-to-failure (TTF) in Fulvestrant users. [file 12967_2018_1734_MOESM1_ESM.docx]

**Table S1. Multivariate COX regression analysis for the risk factors for progression (PFS) and time-to-failure (TTF) in Fulvestrant users.**

|  |  | **PFS** | |  | **TTF** | |
| --- | --- | --- | --- | --- | --- | --- |
| **Covariates** | **Level** | **Hazard ratio (95% CI)** | **p-value** |  | **Hazard ratio (95% CI)** | **p-value** |
| Fulvestrant usage | First line | Ref |  |  | Ref |  |
|  | Second line | 2.816 (0.894, 8.868) | 0.08 |  | 3.094 (0.994, 9.626) | 0.05 |
|  | ≥ Third line | 3.629 (1.092, 12.059) | 0.04 |  | 3.983 (1.230, 12.898) | 0.02 |
| Age at diagnosis (years) |  | 1.112 (1.001, 1.236) | 0.05 |  | 1.101 (0.996, 1.216) | 0.06 |
| Age at FX usage (years) |  | 0.884 (0.792, 0.986) | 0.03 |  | 0.886 (0.797, 0.984) | 0.02 |
|  | <62 yrs | Ref |  |  | Ref |  |
|  | ≥ 62 yrs | 0.450 (0.169, 1.200) | 0.11 |  | 0.442 (0.180, 1.084) | 0.07 |
| DFS (years)* |  | 1.059 (0.921, 1.216) | 0.42 |  | 1.041 (0.912, 1.189) | 0.55 |
| ER | Negative | - |  |  | - |  |
|  | 1%-50% | Ref |  |  | Ref |  |
|  | 50%-100% | 0.663 (0.256, 1.717) | 0.02 |  | 0.807 (0.330, 1.971) | 0.36 |
|  | Unknown | 0.840 (0.326, 2.167) | 0.72 |  | 1.049 (0.445, 2.474) | 0.48 |
| PR | Negative | Ref |  |  | Ref |  |
|  | Positive | 1.069 (0.405, 2.821) | 0.89 |  | 0.783 (0.344, 1.784) | 0.56 |
| HER2 | Negative | Ref |  |  | Ref |  |
|  | Positive | 2.396 (1.024, 5.604) | 0.04 |  | 1.981 (0.865, 4.538) | 0.10 |
| Nuclear or histological grade | 2 | Ref |  |  | Ref |  |
|  | 3 | 1.599 (0.784, 3.263) | 0.20 |  | 1.120 (0.599, 2.097) | 0.72 |
|  | Unknown | 1.856 (0.877, 3.928) | 0.11 |  | 1.378 (0.725, 2.619) | 0.33 |
| Stage at BC diagnosis | 0/I | Ref |  |  | Ref |  |
|  | II | 1.415 (0.712, 2.812) | 0.32 |  | 1.326 (0.684, 2.574) | 0.24 |
|  | III/IV | 1.971 (1.022, 3.802) | 0.04 |  | 1.944 (1.071, 3.530) | 0.03 |
| Menopause | Natural Menupause | Ref |  |  | Ref |  |
|  | OFS (OFS+surgery) | 0.707 (0.265, 1.887) | 0.49 |  | 0.463 (0.184, 1.162) | 0.10 |
|  | Surgery | 1.108 (0.492, 2.495) | 0.80 |  | 0.800 (0.386, 1.658) | 0.55 |
| Treatment after relapse or metastasis | Radiation | 1.121 (0.572, 2.196) | 0.74 |  | 0.894 (0.455, 1.756) | 0.74 |
|  | Chemotherapy | 1.434 (0.787, 2.612) | 0.24 |  | 1.612 (0.923, 2.815) | 0.09 |
| Metastatic sites | Lymph nodes | 2.863 (1.628, 5.036) | 0.0003 |  | 2.786 (1.615, 4.807) | 0.0002 |
|  | Bone | 1.610 (0.862, 3.007) | 0.14 |  | 1.716 (0.935, 3.150) | 0.08 |
|  | Visceral | 1.155 (0.649, 2.055) | 0.62 |  | 0.991 (0.581, 1.689) | 0.97 |
