# Supplementary figures and images for: Clinical and genetic risk factors for Fulvestrant treatment in post-menopause ER-positive advanced breast cancer patients
Source: J Transl Med. 2019 Jan 15;17:27. doi: 10.1186/s12967-018-1734-x (PMC6334389; doi:10.1186/s12967-018-1734-x)

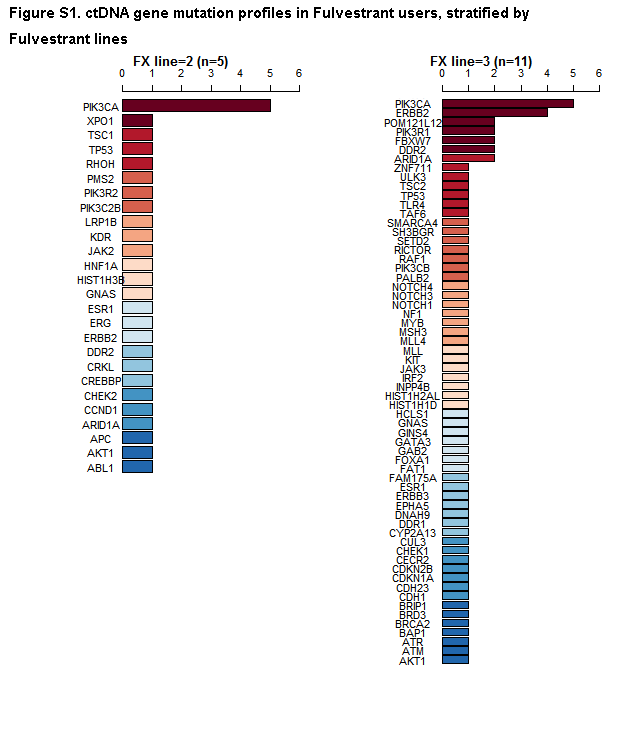

Supplement: Supplementary file 2 — Additional file 2: Figure S1. ctDNA gene mutation profiles in Fulvestrant users, stratified by Fulvestrant lines, second-line users (A), and third or higher-line users (B). Dark red represents the most common mutated genes and dark blue represents the rarest mutations. If the mutated genes appeared at the same frequency, they are ranked in alphabetic order. [file 12967_2018_1734_MOESM2_ESM.tif]
